# The association between time definition of reintubation and patient outcomes in critically ill patients: several topics should be noticed

**DOI:** 10.1186/s13054-023-04680-7

**Published:** 2023-10-16

**Authors:** Libing Jiang, Hongyu Zhang

**Affiliations:** Department of Emergency Medicine, Second Affiliated Hospital, Zhejiang University School of Medicine, Key Laboratory of The Diagnosis and Treatment of Severe Trauma and Burn of Zhejiang Province, Zhejiang Province Clinical Research Center for Emergency and Critical Care Medicine, Hangzhou, 310009 China

To the editor,

We read with great interest the recent study by Dr. Tanaka et al. [1], in which they explore the association between reintubation and patient outcomes as well as the consequences of the time elapsed between extubation and reintubation on patient outcomes. Finally, 1849 patients underwent reintubation were included, and they reported that reintubation would result in higher in-hospital and intensive care unit (ICU) mortality. In addition, they found the patients who were reintubated between 72 and 96 h after extubation had the highest mortality rates. This is a significant topic. We would like to add some comments.

First, as the authors mentioned mechanical ventilation procedures, especially the decision to extubate or reintubate, were determined at the discretion of the clinicians. This is very important for the robustness of the results. Although the authors described the weaning protocol, the decision of extubation and reintubation is still relatively subjective. And they did not evaluate the levels of adherence to the reporting guideline. Apart from that, they did not report the reintubation algorithm. The decision to reintubate can vary widely from doctor to doctor. Thus, the characteristics of each ICU included in this study should be considered in the multivariables analysis.

Second, as we all know, conventional oxygen therapy, high-flow conditioned oxygen therapy, and noninvasive ventilation (NIV) are three noninvasive methods to increase oxygenation after extubation. Overall, high-flow conditioned oxygen therapy and NIV are superior to conventional oxygen therapy for preventing reintubation and postextubation respiratory failure. However, there are no definitive conclusions to date about whether the use of high-flow conditioned oxygen therapy after extubation reduces the risk of reintubation compared with high-flow nasal oxygen [2]. Meanwhile, different strategies may vary the outcomes. For example, in Arnaud et al.’s study, they found the use of high-flow nasal oxygen with NIV immediately after extubation significantly decreased the risk of reintubation compared with high-flow nasal oxygen alone in mechanically ventilated patients at high risk of extubation failure [3]. Thus, it is better to describe the specific oxygen therapy modalities used in this study.

Third, the indications of tracheostomy may vary among different ICUs. Patients receiving tracheostomy during the first mechanical ventilation episode were excluded. But for these patients, different physicians in each hospitals might make different decisions. In other words, these patients who received tracheostomy may extubated successfully in other hospitals. This could bias the results.

## Data Availability

Not applicable.
